# Pediatric Synovial Sarcoma in the Retropharyngeal Space: A Rare and Unusual Presentation

**DOI:** 10.1155/2015/587386

**Published:** 2015-01-06

**Authors:** Sanjay Vaid, Neelam Vaid, Sanjay Desai, Varada Vaze

**Affiliations:** ^1^Head and Neck Imaging Division, Star Imaging and Research Center, Pune 411001, India; ^2^Department of Otorhinolaryngology, KEM Hospital, Pune 411011, India

## Abstract

Synovial sarcomas in the head and neck are extremely rare tumors, especially in the pediatric population. 3–5% of synovial sarcomas occur in the head and neck region displaying varied imaging and histopathological features resulting in frequent misdiagnosis. These tumors have a poor prognosis; hence early diagnosis and accurate classification based on imaging, histopathology, and immunohistochemistry are critical for prompt treatment. To the best of our knowledge, imaging findings of pediatric retropharyngeal lipomatous synovial sarcoma have not been reported to date in English medical literature. We report, for the first time, a rare case of retropharyngeal lipomatous synovial sarcoma in a ten-year-old child and discuss the case-specific imaging findings in our patient using magnetic resonance imaging and computed tomography.

## 1. Introduction

Synovial sarcomas are well known clinical entities occurring in para-articular locations close to synovial linings, tendon sheaths, and bursae. These tumors are uncommon in the head and neck but cases have been reported in the adult population involving the oral cavity, sinonasal tract, submandibular area, parotid gland, temporal/infratemporal regions, cheek, supra-auricular scalp, maxillary/submaxillary regions, mandible, and nasolabial regions [1]. It is important to make an early diagnosis as these tumors have a poor prognosis and prolonged insidious clinical course. Most cases present late due to symptoms attributable to mass effect on adjacent critical structures after having attained a large size [2]. High resolution multiplanar CT and MRI demonstrate the local infiltration as well as extension to adjacent structures and play an important role in characterizing the internal architecture based on typical imaging features.

## 2. Case Presentation

**Figure 1:**
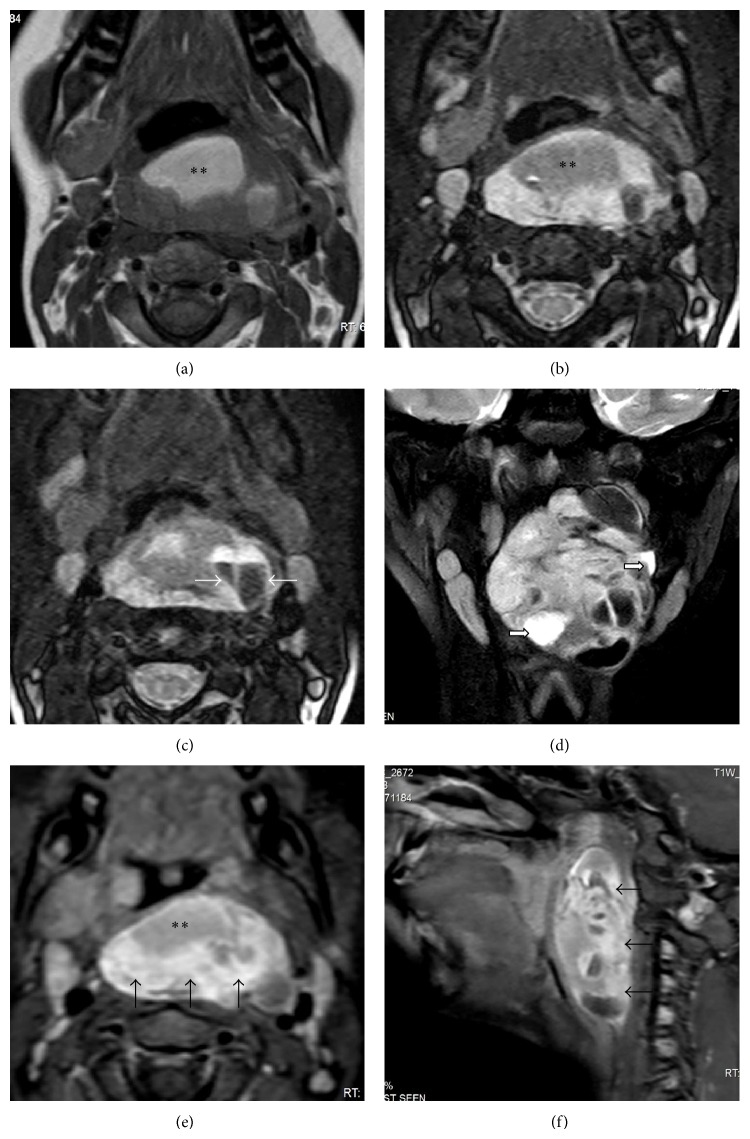
T1W axial (a), STIR axial ((b), (c)), and coronal (d) images showing a large complex signal retropharyngeal mass with a large lipomatous component (asterisk) and multiple fluid-fluid levels (white arrows) and scattered cystic components (block arrows). Postcontrast fat saturated axial (e) and sagittal (f) images reveal dense heterogeneous enhancement within the soft tissue component of the mass (black arrows).

A ten-year-old male child presented with dysphagia, breathlessness, disturbed sleep, and snoring for two months. There was no history of fever or throat pain. Clinical examination of the oral cavity revealed a large smooth bulge on the posterior pharyngeal wall with intact overlying mucosa. The swelling was firm, nontender and its inferior margin could not be identified. No neck nodes were palpable on clinical examination. The patient was referred for preoperative radiological evaluation. A contrast-enhanced MRI examination and a noncontrast low-dose CT scan were performed. The imaging studies revealed a large well-defined mass with smooth margins and a complex signal appearance in the midline retropharyngeal space measuring approximately 4.8 × 1.5 cm in maximum transverse and anteroposterior dimensions and extending superoinferiorly for approximately 6.6 cm (Figure 1). The lesion showed multiple well-defined loculi, cystic (with fluid levels) as well as solid components, and a large lipomatous component (confirmed by typical imaging appearance on T1-weighted and fat saturated T2-weighted MR sequences and CT attenuation values indicative of adipose tissue). The solid nonlipomatous components of the lesion showed homogeneous postcontrast enhancement. No calcification or hemorrhagic foci were noted. Marked mass effect was noted on the nasopharynx, oropharynx, hypopharynx, and supraglottic larynx which were anteriorly displaced but appeared intrinsically normal. Bilateral multilevel cervical lymphadenopathy was noted; however, all the lymph nodes were oval in shape with intact fatty hilum and were subcentimeter in size. These were interpreted radiologically as reactive lymph nodes with no suspicion of malignancy.

Based on the above imaging appearances and age of the patient, the differential diagnosis included teratoma, dermoid cyst, and a fat containing mesenchymal tumor.

**Figure 2:**
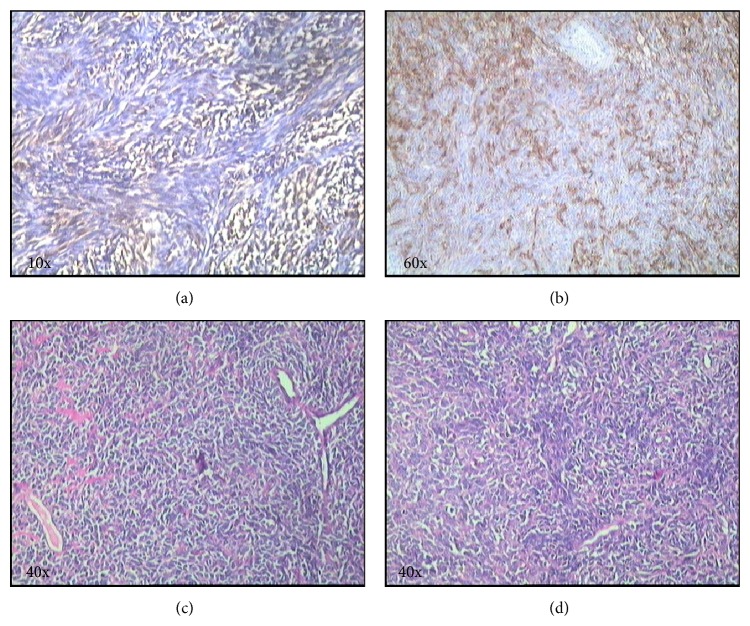
Immunohistochemistry and histopathological sections reveal tumor cells positive for bc12 (a) and focally positive for EMA (b), and the tissue shows polygonal to spindle shaped cells arranged in whorls and storiform pattern ((c), (d)) on haematoxylin and eosin staining.

The patient underwent a wide local excision of the mass under general anesthesia via a transoral approach. The postoperative period was uneventful. Histopathological examination of the operative specimen showed polygonal to spindle shaped cells arranged in whorls and storiform pattern (Figure 2). Scattered foci of adipose tissue were identified within the stroma of the tumor which did not show abnormal features or evidence of malignancy. On immunohistochemistry, the tumor cells were positive for bc12 and focally positive for Mic2 and EMA. The cells were negative for CD34, S100, desmin, and PR.

A diagnosis of monophasic synovial sarcoma was made based on the above findings. The patient subsequently received radiation therapy over a period of five months and has been advised regular six-monthly follow-up at our ENT outpatient department. There was no evidence of local or systemic recurrence on the PET-CT scan carried out three months after completion of radiation therapy.

## 3. Discussion

The retropharyngeal space is situated posterior to the pharynx and esophagus and anterior to the prevertebral muscles. The detailed anatomy of this neck space and the extensive differential diagnosis of pathologies have been discussed extensively in existing medical literature [3]. Benign lipomas in the head and neck region are uncommon [4], and fat containing malignant tumors, that is, liposarcomas, in this region are rare, accounting for only 1.8–6.3% of cases. Cases of liposarcoma in the retropharyngeal space are even more uncommon and, to the best of our knowledge, only six cases have been reported previously, that too only in the adult population [5]. Imaging plays an important role in the preoperative assessment of these cases as the lipomatous component of the mass and the enhancing soft tissue components can be easily identified and confirmed using Hounsfield values on CT and the signal pattern specific to adipose tissue on T1-weighted and T2-weighted fat suppressed MRI sequences [6]. Head and neck region synovial sarcomas are rare tumors in the pediatric population and occur due to malignant degeneration of pluripotential primitive mesenchymal cells which can be located at a location remote from synovial tissue. These tumors appear to originate primarily from the paravertebral connective tissue spaces, presenting later as retropharyngeal or parapharyngeal masses, with the hypopharynx being the commonest site in the head and neck [2, 6]. Three histological variants have been described: the classical biphasic type, the monophasic fibrous type, and the monophasic epithelial type [2]. None of the synovial sarcomas described to date in medical literature have reported the presence of adipose tissue within the tumor. The recommended treatment is wide local excision with negative margins followed by postoperative radiotherapy to improve local control rates [6]. In our patient the tumour was removed piecemeal and, hence, disease-free margins could not be documented. Once the histopathology of the tumor was confirmed, postoperative radiotherapy was advised for treating any possible residual tumor tissue. Studies conducted in the past [7, 8] indicate that radiotherapy significantly reduces local recurrence in patients with suspected residual tumour. Hence, this treatment protocol was used in the management of our patient. Adjuvant chemotherapy may be added to the treatment to delay or prevent distant metastases but it was not included in the treatment protocol in this case. In retropharyngeal synovial sarcomas, CT examinations are useful to show calcifications (seen in approximately 30% of all cases) which are indicative of better survival rates [6]. On MRI imaging, synovial sarcomas reveal a tumor of intermediate intensity on T1-weighted sequences and of variable intensity on T2-weighted sequences, with heterogeneous enhancement after injection of contrast material [6, 9]. Foci of hemorrhage, fluid-fluid levels, and cyst formations are commonly seen. Our case did not show evidence of hemorrhage but the lesion showed fluid-fluid levels, cyst formation, and large amount of adipose tissue. Metastatic lymph node involvement occurs in 12.5% of the cases of head and neck synovial sarcomas [6]. As is seen in other types of sarcoma, pulmonary metastases are common in synovial sarcomas [2]. However, our patient had a normal preoperative chest X-ray and postoperative CT scan of the thorax.

## 4. Conclusion

This case report highlights the imaging findings in a child with a lipomatous synovial sarcoma of the retropharyngeal space. There were no specific imaging criteria to make a preoperative diagnosis which was confirmed only postoperatively by histopathology and immunohistochemistry. A complex signal intensity mass on MRI (even those with well-defined smooth margins) in the retropharyngeal space with solid/cystic components, fluid-fluid levels, and heterogeneous postcontrast enhancement indicates a high possibility of a malignant pathology, even in children. Presence of adipose tissue in such a complex mass further raises the index of suspicion for malignancy and all such lesions should be treated aggressively to lower incidence of metastatic cervical lymphadenopathy and distant metastases.
